# Perception of value from food delivery apps: A data report

**DOI:** 10.3389/fpsyg.2022.973724

**Published:** 2022-09-16

**Authors:** Aini Aman, Omkar Dastane, Muhammad Rafiq

**Affiliations:** ^1^Faculty of Economics and Management, Universiti Kebangsaan Malaysia, Selangor, Malaysia; ^2^UCSI Graduate Business School, UCSI University, Kuala Lumpur, Malaysia

**Keywords:** perceived value, perception, consumer psychology, online food delivery (OFD), food delivery apps (FDAs), mobile apps

## Introduction

The food delivery sector has expanded from takeout to everything, adding billions of dollars in revenue collection during the previous 5 years. China leads in overall food delivery revenue in 2021, with $27.3 billion. As the largest meal delivery app in the world based on revenue and use, Meituan contributed $15 billion to this total. The meal delivery market is anticipated to reach $320 billion in 2029 (STATISTA, 2022). Due to the coronavirus pandemic, it grew at its fastest rate in 5 years in 2020. However, this exponential rise has also intensified rivalry in an already tough business. FDA firms face obstacles such as low customer retention, a high churn rate, discount-driven sales, low profits, and low app engagement (AppAnnie, 2022). This necessitated an understanding of strategies to increase the continued use of FDA and repurchase intent.

Mobile commerce, which is a competitive market, which therefore requires businesses to offer the right and attractive value proposition based on the perception of consumers to gain sustainable competitive advantage (Leppäniemi et al., 2017; Aman et al., 2022). In the wake of (Zeithaml, 1988) pioneering description of customer perceived value (CPV), other scholars have added that the initial one-dimensional approach has been broadened to include multidimensional and higher-order CPV dimensions (see Sheth et al., 1991; Holbrook, 1994). Additionally, the same was evaluated for several sectors and marketplaces (e.g., Haba, 2019; Tandon et al., 2021). When it comes to modeling CPV for mobile customers, however, the literature is scant and places a heavy emphasis on technological concerns, leaving out other crucial factors (Dastane et al., 2020). On a more significant note, a substantial amount of research has highlighted what consumers perceive as the value of mobile commerce; however, these studies are either dominated by technical aspects or largely based on the foundation of traditional models of perceived value to the mobile shopping context. Therefore, it is constantly necessary to rethink, reinvestigate, and re-conceptualize perceived value in order to determine what customers perceive as valuable. Overall, this encapsulates the requirement to comprehend and describe perceived value from mobile commerce since identifying and investigating perceived value dimensions might be the key to resolving industry difficulties, given the high cost of conversion and user acquisition. In addition, it is necessary to analyze beyond the technical elements of M-commerce to ensure that M-businesses can deliver a compelling value proposition. According to Zauner et al. (2015), the classic CPV components are interdisciplinary in nature, with the majority originating from psychology and economics. Consequently, it is vital to conceptualizing mobile CPV. It is obvious that a substantial and expanding body of research has explored the needs of mobile consumers, particularly in terms of maximizing the advantages and minimizing the costs of mobile shopping; yet the aspects have not been investigated through suitable dimensions.

CPV provides a robust framework for comprehending consumer perception by merging time-tested concepts on the perception of value in consumer behavior research. Traditionally, perceived value has been seen to be subjective, the consequence of a cost-benefit tradeoff, and connected to the consuming perception (Holbrook, 1994). In addition, the CPV has been effectively applied to the online environment to acquire insights into the value consumers derive from digital and virtual worlds (Haba, 2019; Tandon et al., 2021). Therefore, we utilized the CPV to contribute to an ongoing study on consumer behavior in the FDA context. Recently, the focus of research has shifted to mobile app experience to create methods to lure customers to visit online companies and influence their purchase propensities. Little is known in the context of mobile apps, particularly FDAs, regarding why users choose one app over another and the perceived value they derive (Tandon et al., 2021).

The objective of this data report is to document a dataset related to consumer perception. To be specific, the data set presents consumer perceptions of value in the context of using FDAs. This data report is valuable in several aspects. Firstly, it may be used as a guide to figure out what individuals want from their buying experience through FDA. Secondly, this data is beneficial for all parties involved, especially for FDA service providers, digital marketers, and mobile advertisers. Thirdly, this data may be used to identify what motivates consumers to buy from FDA again and again. Finally, researchers may utilize this information to create a cross-country comparison model. Most importantly, the extant literature in this context is dominant in theories related to technology adoption, theory of planned behavior, and theory of reasoned action. This data report presents data collected based on the grand theory of customer perceived value which has its roots in the consumers' perception and is therefore appropriate for social science and marketing research.

## Method

### Procedure

The study used a cross-sectional quantitative approach. Individuals are defined as working professionals mainly between the ages of 21 and 50 who must be employed and have a smartphone with internet access as the unit of analysis. The samples were collected using a non-probability sampling approach. Because of the crucial pre-determined criteria of 1. age 2. employability and 3. availability of smartphones with the internet, purposive sampling, and snowball sampling were utilized. A Google Form was used to create an online questionnaire, and the link was shared *via* emails, messaging applications (such as Whatsapp, WeChat, and others), and professional social networking sites. The data was collected during April and May of 2020. As a result, 319 suitable replies were collected for future investigation. The sample size of 319 was larger than the minimum sample size recommended by G Power (v.3.1.9.7) (Hair et al., 2014). Individual demographic characteristics, functional value, conditional value, social value, epistemic value, and intention to continue FDA are all covered by the questionnaire. A total of 17 items derived from previous research are used to assess the relevant intention to adopt m-commerce characteristics, with all items using a five-point Likert scale ranging from “1” (strongly disagree) to “7” (strongly agree).

### Survey questionnaire

The questionnaire contains 17 items that measure the relevant consumption value aspects leading to a continuance intention toward FDA. The perceived value dimensions included are functional value (monitory aspects, four items) (Tandon et al., 2021), conditional value (situational aspects, three items), social value (prestige-related aspects, three items) (Dastane et al., 2020), and epistemic value (visibility aspects, three items) (Sheth et al., 1991). In addition, individual demographic characteristics and frequency of monthly FDA usage are covered by the questionnaire. The continuance intention was measured with relevant four items. The detailed questionnaires were made available along with the data set and can be retrieved from the link given in the section on data availability.

## Data description

### Data characteristics

There were 319 valid responses in total. The questionnaire data is compiled into a raw data file that is attached as a supplemental document to this main article. Gender, age, ethnicity, the highest level of education, income, marital status, and FDA consumption frequency in a month are among the demographic factors shown in Table 1. Frequencies and percentages were used to analyze the data.

**Table 1:** Demographics of participants (*N* = 319).

**Variable**	**Category**	**Frequency**	**Percentage (%)**
Gender	Male	186	58.5
	Female	132	41.5
Age	Below 21	7	2.2
	21 to 30	43	13.5
	31 to 40	123	38.7
	41 to 50	109	34.3
	Above 50	36	11.3
Ethnicity	Malay	39	12.3
	Chinese	232	73
	Indian	41	12.9
	Others	6	1.9
Education	Diploma	78	24.5
	Degree	151	47.5
	Masters	54	17
	Others	35	11
Marital status	Single	98	30.8
	Married	102	32.1
	Married with Children	118	37.1
Income	Below RM 2500	37	11.7
	RM 2501 to RM 5000	71	22.3
	RM 5001 to RM 7500	65	20.4
	RM 7501 to RM 10,000	94	29.6
	RM 10,000 and above	51	16
Monthly FDA usage frequency	Once	12	3.7
	2 to 5	119	37.4
	6 to 10	117	36.8
	10 and above	49	15.4

### Measurement model

The absolute fit, incremental fit, and parsimonious fit parameters were used to assess the study's measurement model's fitness as recommended by Hair et al. (2014) and Rafiq et al. (2022). With an RMSEA value of 0.069 and RMR of 0.62, the measurement model evidently obtained a good absolute fit as recommended by Hair et al. (2014), Ibrar et al. (2017), and Rafiq et al. (2022), while a Chisq/df value of 2.501 indicated a parsimonious match. With a CFI of 0.963, TLI of 0.954, and NFI of 0.940, the model also obtained an incremental fit. The GFI value was 0.906, which was only acceptable if the model matched the data efficiently (Wai et al., 2019).

### Validity assessment

Table 2 shows the results of the reliability and validity investigation. Cronbach's alpha, compositive reliability, and average variance extracted (AVE) are markers for assessing construct reliability (Keong, 2019). The components were determined to be above the 0.70 criteria, showing internal consistency reliability (Hamzah et al., 2022), and convergent validity was demonstrated with an AVE value more than 0.5. Meanwhile, discriminant validity is investigated using the Fornell- Larcker criteria (see Table 2), which involves comparing the AVE of each construct to the squared inter-construct correlation of the same and other constructs in the model (Ren et al., 2019). Collinearity issues are also investigated by looking at the variance inflation factor (VIF), which should be less than 5 as indicated (Hamzah et al., 2022). All constructions are well below the threshold of 5, suggesting that there are no difficulties with multicollinearity. The results are included in Table 2.

**Table 2:** Reliability and validity.

	**Items**	**Loadings**	**Cronbach's Alpha**	**CR**	**AVE**	**Tolerance**	**VIF**	**MSV**	**MaxR**	**EV**	**FV**	**SV**	**CV**	**CI**
Functional value	FV1	0.831	0.878	0.82	0.7	0.381	2.63	0.436	0.912	0.872				
	FV2	0.836												
	FV3	0.829												
	FV4	0.715												
Conditional value	CV1	0.792	0.876	0.88	0.7	0.509	1.96	0.637	0.82	0.621	0.804			
	CV2	0.885												
	CV3	0.835												
Social value	SV1	0.805	0.924	0.93	0.81	0.491	2.04	0.507	0.949	0.538	0.73	0.901		
	SV2	0.946												
	SV3	0.945												
Epistemic value	EV1	0.816	0.902	0.9	0.76	0.635	1.58	0.621	0.884	0.46	0.788	0.636	0.838	
	EV2	0.901												
	EV3	0.895												
Continuance intention	CI1	0.843	0.908	0.91	0.72	-	-	0.637	0.915	0.66	0.798	0.712	0.692	0.847
	CI2	0.858												
	CI3	0.892												
	CI4	0.79												

### Structural model

Functional value, conditional value, social value, and epistemic value are the independent factors investigated, while continuation intention toward FDAs is the dependent variable. The structural model was deemed an excellent match since it met both absolute (RMEA = 0.069, GFI = 0.906) and incremental (CFI = 0.963, TLI = 0.954, NFI = 0.940) fit criteria. The findings of path analysis corroborate the strength and importance of the relationships between the constructs. The path analysis is then conducted, and the results are as follows. The function value had a positive and significant influence on continuance intention with a beta value of 0.369 and with a *p*-value of 0.000. The impact of conditional value was also recorded as positive with a beta value of 0.153 and at a significance level of *p* < 0.05 with a *p*-value of 0.043. Next, social value also demonstrated an impact on continuance intention of 0.213 with a *p*-value of 0.000 and therefore this impact is considered as significant as well. Lastly, the epistemic value also showed a positive (beta value 0.246) and significant impact (*P* = 0.000) on continuance intention.

## Discussion

### Implications

The report includes data from multiple utilities. In the area of FDA and OFD, the existing research is dominated by studies on technology adoption rather than continuation intent. In addition, ideas associated with information systems dominate these investigations rather than consumer behavior or psychological theories. By developing enticing value propositions, FDA business managers seek novel approaches to increase the offering's worth. To make applications appealing and user-friendly, app developers are searching for customer-centric characteristics. Digital marketers want direction to guarantee proper marketing to increase market share and client loyalty. This data report is beneficial in several ways. First, it may be used as a guide to determine what consumers want from their FDA purchasing experience. Second, this information is advantageous for all stakeholders, particularly FDA service providers, internet marketers, and mobile advertising. Thirdly, this information may be utilized to determine what encourages consumers to repeatedly purchase from FDA. Finally, researchers might use this data to develop a cross-national comparison model.

### Limitations

The data report is based on a model that considers four factors of perceived value based on existing theories in the area. Future research can undertake qualitative inquiries to find distinctive aspects especially associated with FDA. In addition, it is urged that data on various mediators and moderators be gathered in the future. Future research may also concentrate on collective data gathered through random sampling to ensure more generalizability of outcomes. The data is obtained in Malaysia; therefore, it is difficult to apply the findings to other nations, limiting the generalizability of the conclusions.

## Data availability statement

The datasets presented in this study can be found in online repositories. The names of the repository/repositories and accession number(s) can be found in the article.

## Ethics statement

Ethical review and approval was not required for the study on human participants in accordance with the local legislation and institutional requirements. The patients/participants provided their written informed consent to participate in this study.

## Author contributions

AA: re-conceptualization, methodology, supervision, and review. OD: conceptualization, data collection, methodology, and writing—original draft. MR: methodology, formal analysis, and writing—review. All authors contributed to the article and approved the submitted version.

## Funding

This work is funded under grant number UKM-TR-003, Universiti Kebangsaan Malaysia.

## Conflict of interest

The authors declare that the research was conducted in the absence of any commercial or financial relationships that could be construed as a potential conflict of interest.

## Publisher's note

All claims expressed in this article are solely those of the authors and do not necessarily represent those of their affiliated organizations, or those of the publisher, the editors and the reviewers. Any product that may be evaluated in this article, or claim that may be made by its manufacturer, is not guaranteed or endorsed by the publisher.
